# A Hydroxyquinoline‐Based Unnatural Amino Acid for the Design of Novel Artificial Metalloenzymes

**DOI:** 10.1002/cbic.202000306

**Published:** 2020-07-17

**Authors:** Ivana Drienovská, Remkes A. Scheele, Cora Gutiérrez de Souza, Gerard Roelfes

**Affiliations:** ^1^ Stratingh Institute for Chemistry University of Groningen Nijenborgh 4 9747 AG Groningen The Netherlands

**Keywords:** biocatalysis, hybrid catalysts, metalloenzymes, noncanonical amino acids, protein design

## Abstract

We have examined the potential of the noncanonical amino acid (8‐hydroxyquinolin‐3‐yl)alanine (HQAla) for the design of artificial metalloenzymes. HQAla, a versatile chelator of late transition metals, was introduced into the lactococcal multidrug‐resistance regulator (LmrR) by stop codon suppression methodology. LmrR_HQAla was shown to complex efficiently with three different metal ions, Cu^II^, Zn^II^ and Rh^III^ to form unique artificial metalloenzymes. The catalytic potential of the Cu^II^‐bound LmrR_HQAla enzyme was shown through its ability to catalyse asymmetric Friedel‐Craft alkylation and water addition, whereas the Zn^II^‐coupled enzyme was shown to mimic natural Zn hydrolase activity.

## Graphical abstract

**Expanding biotransformations**: Several metal‐binding noncanonical amino acids have been incorporated into protein scaffolds to create artificial metalloenzymes. We introduced 2‐amino‐3‐(8‐hydroxyquinolin‐3‐yl)propanoic acid (HQAla) into lactococcal multidrug‐resistance regulator (LmrR) and complexed it with several transition metal ions such as Cu^II^, Zn^II^ and Rh^III^ to showcase its catalytic potential in a variety of different reactions.

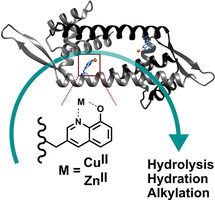

Metalloproteins represent more than one‐third of natural enzymes, catalysing many of the important chemical reactions that sustain life.^[1]^ This has inspired the creation of artificial metallo‐enzymes, which are hybrids of proteins and synthetic transition metal complexes, ultimately aiming to develop biocatalysts for reactions that are new to nature.^[2]^

Synthetic transition metal complexes have been incorporated into protein scaffolds using different methods to bind the metal‐ligand complexes.^[3]^ A recent approach to introduce metal catalysts involves noncanonical amino acids (ncAAs), either serving as the ligand which directly chelates the metal, or by binding an external metal‐binding ligand. The ncAAs are introduced using expanded genetic code methods, especially stop codon suppression.^[4]^

Several metal‐binding ncAAs have been incorporated into proteins *in vivo* by expanded genetic code methodology to create artificial metalloenzymes.^[5]^ For example, the amino acid analogue of the bipyridine ligand, (2,2’‐bipyridin‐5‐yl)alanine (BpyA), has been incorporated into different biomolecular scaffolds to bind different bivalent metals and catalyse a variety of reactions when complexed with Cu^II^.^[6]^ Likewise, the incorporation of noncanonical N‐methylhistidine and 3,4‐dihydroxyphenylalanine into metallo‐enzymatic scaffolds has been shown to both boost enzymatic turnover, and facilitate reutilisation of an alcohol dehydrogenase active site to bind Zn^II^, respectively.^[7]^

The metal ligand 8‐hydroxyquinoline (HQ) is one of the earliest analytical reagents and has been shown to bind >20 transition metals.^[8]^ More recently, the strong N−O bidentate binding mode of HQAla has been used for a variety of Cu^II^, Zn^II^ and Rh mediated reactions, highlighting the versatility of HQ.^[9]^ The ncAA that employs 8‐hydroxyquinoline as its functional group, 2‐amino‐3‐(8‐hydroxyquinolin‐3‐yl)propanoic acid (HQAla) (Figure 1), was first incorporated as unnatural amino acid into the Z‐domain protein. Here it was used as a fluorescent probe and to bind heavy metals for crystallography.^[10]^ The constitutional isomer of this ncAA, 2‐amino‐3‐(8‐hydroxyquinolin‐5‐yl)propanoic acid, has been described and used in protein electron transfer and Zn^II^ sensing *in vitro* and *in vivo*.^[11]^

**Figure 1:**
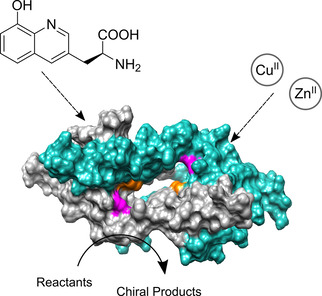
Schematic representation of the proposed design for novel artificial metalloenzymes. Surface representation of the LmrR protein scaffold (PDB ID: 3F8B) with the positions chosen for the incorporation of HQAla highlighted in orange (V15) and magenta (M89).

So far, expansion of the genetic code with HQAla has not resulted in designer biocatalysts. Here, we introduce HQAla into lactococcal multidrug‐resistance regulator (LmrR)^[12]^ and complex it with a variety of transition metal ions such as Cu^II^, Zn^II^ and Rh^III^. The catalytic propensity of the Cu^II^ bound enzymes was demonstrated in a vinylogous Friedel‐Crafts alkylation and an enantioselective hydration reaction. The Zn^II^ bound artificial metalloenzyme was shown to hydrolyse amide bonds.

The artificial metalloenzymes presented in this study were prepared by using amber stop codon methodology, with a plasmid carrying the orthogonal tRNA‐synthetase/tRNA pair specific for the incorporation of HQAla (pEVOL‐HQAla).^[10]^ HQAla was synthesised according to a previously reported method with a few minor improvements.^[10]^ The positions M89 and V15 of LmrR were selected for the incorporation of HQAla, as these positions have been previously shown to allow efficient incorporation of ncAAs.[^6e^, ^6f^, ^13^] Both residues are pointing towards the inside of the hydrophobic pore of the protein, with M89 located at the far edge and V15 more to the middle of the pore. The M89 position was shown to be the optimal position for studies with BpyA,[^6e^, ^6f^] whereas position V15 has been recently described as the optimal position for *p*‐aminophenylalanine incorporation.^[13]^

The pEVOL‐HQAla plasmid and a pET17b plasmid containing either LmrR_V15TAG or LmrR_M89TAG constructs were cotransformed into *Escherichia coli* C43(DE3). The LmrR variant used in this study contains two mutations, K55D and K59Q, which are introduced to remove the DNA binding ability of LmrR, and a C‐terminal Strep‐Tag.^[14]^ After addition of HQAla to the medium, the cells were induced to produce LmrR_V15HQAla or LmrR_M89HQAla (Figure S1 in the Supporting Information). The expression yields were 18–22 mg/L for LmrR_V15HQAla and 4–10 mg/L for LmrR_M89HQAla. The lower yield of LmrR_M89HQAla is mainly attributed to faulty translation, that is, failure to suppress the stop codon UAG, resulting in truncated LmrR(1–88). The incorporation of HQAla was confirmed by electrospray ionisation mass spectrometry (ESI‐MS; Figure S2). The quaternary structure of LmrR was studied by analytical size‐exclusion chromatography to determine the effect of unnatural amino acid incorporation. It was found that the structure was preserved and the proteins were eluted as single peaks at 11.4 (±0.1) mL, which represents a molecular weight of approximately 30 kDa, consistent with dimeric LmrR (Figure S3).

The ability of LmrR_V15HQAla and LmrR_M89HQAla to bind metal salts was studied by UV‐Vis titrations, measuring the change in the UV‐Vis absorption spectrum upon addition of the metal salts. The titrations were performed with Cu(NO_3_)_2_, Zn(NO_3_)_2_, Cp*RhCl_2_, RhCl(COD), Rh_2_(AcO)_4_ and Rh_2_Cl_2_(CO)_4_. Titration of Cu(NO_3_)_2_ to LmrR_V15HQAla or LmrR_M89HQAla caused hypochromic shifts to the ligand‐centred (LC) charge transfer band at 249 and to a lesser extent the LC band at 320, arising from π–π* and n–π* transitions respectively. Simultaneously, hyperchromic shifts of ligand‐to‐metal charge transfer (LMCT) transitions at 269 nm and to a lesser extent at ∼390 nm were observed (Figures 2ab and S4).^[15]^ These shifts are in agreement with complex formation of metals to the quinoline moiety involving deprotonation of the phenolic group.^[16]^ When approximately one equivalent of Cu(NO_3_)_2_ with respect to the protein monomer was added, no more changes were observed in the spectrum. Furthermore, LmrR without HQAla showed no changes in absorption upon addition of Cu(NO_3_)_2_ (Figure 2c). This suggests that the HQAla residue is the preferred Cu^II^ binding site, albeit that some unspecific binding to other parts of the protein, which is not detectable by UV‐Vis measurements, cannot be excluded. Using the same method, Zn(NO_3_)_2_ and the rhodium complexes were titrated against LmrR_V15/M89HQAla and LmrR (Figures S4 and S5). Complexing of HQala with Zn(NO_3_)_2_ and Cp*RhCl_2_ caused similar spectral changes until one equivalent of metal was added, indicating binding accompanied by deprotonation. Again, no changes in absorption were observed when titrating either metal against LmrR, indicating binding of Zn^II^ and rhodium‐bound cyclopentadienyl to the HQ moiety in LmrR specifically. Rh_2_(AcO)_4_ and Rh_2_Cl_2_(CO)_4_ did not seem to bind LmrR or HQAla, as the absorption spectra of both LmrR_V15HQAla and LmrR did not change upon titration with these rhodium salts. RhCl(COD) behaved different, altering the absorbance of LmrR_V15HQAla and LmrR beyond the addition of 1 equivalent, and was therefore thought to bind unspecifically to the scaffold. Taken together, the versality of HQAla to bind metal ligands proved to be effective in capturing Cu^II^, Zn^II^ or Cp*Rh^III^ into the dimeric LmrR scaffold.

**Figure 2:**
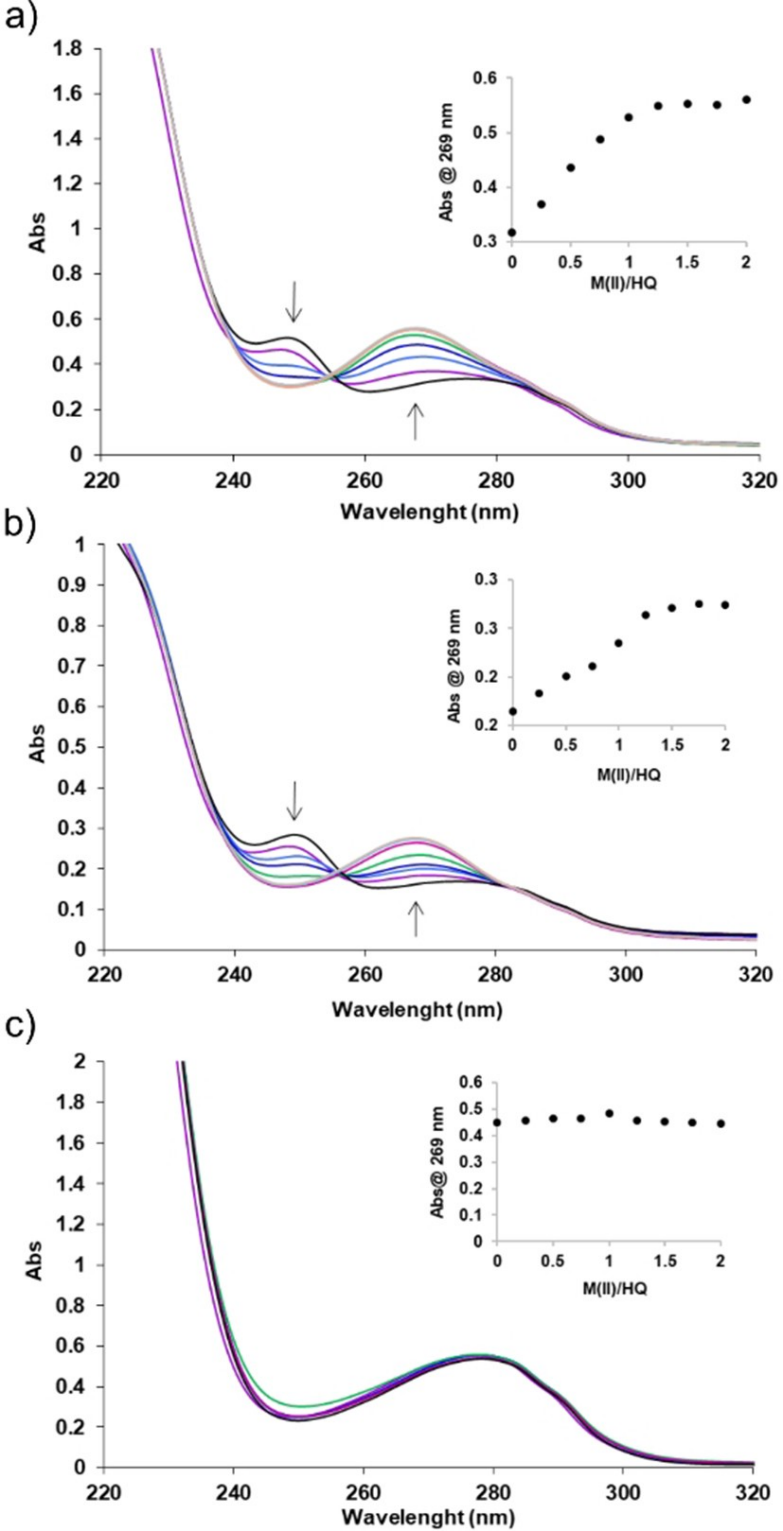
UV‐visible titrations of Cu(NO_3_)_2_ with a) LmrR_V15HQAla, b) LmrR_M89HQAla and c) LmrR. Insets: plots of the absorbance at 269 nm as a function of the equivalents of Cu(NO_3_)_2_ added.

The catalytic potential of these novel artificial metalloenzymes was studied in several reactions. Previous functionalisation of LmrR with a covalently attached Rh catalyst was successful in the catalytic hydrogenation of CO_2,_ ^[17]^ whereas the water stable Cp*Rh complex was used to accelerate aromatic C−H activation when bound by streptavidin.^[18]^ However, testing LmrR_V15/M89HQAla complexed with Cp*Rh for the same aromatic C−H activation did not give rise to detectable product formation, most likely due to the limited number of free coordination sites when Cp*Rh is bound to HQAla.

Zn^II^ serves as the catalytic metal in numerous different hydrolases.^[19]^ Therefore, we decided to probe the hydrolytic potential of LmrR_HQAla_Zn^II^ on five different substrates. First, the hydrolysis of ester bonds was studied by using two model substrates: *p*‐nitrophenyl acetate and *p‐*nitrophenyl butyrate. Although LmrR_HQAla_Zn^II^ hydrolyses these substrates with rates 3–3.5 times higher than the uncatalysed reaction, the fact that unbound Zn(NO_3_)_2_ with LmrR yielded similar rate accelerations indicated that the specific active site was not required for these reactions to proceed. The hydrolysis of *p*‐nitrophenylphosphate was tested, however no hydrolysis was observed, with or without the metal bound.

Next, the ability of LmrR_V15/M89HQAla_Zn^II^ to hydrolyse amide bonds was examined using substrates **1** and **2** (Figure 3). Both of these substrates are small peptides with two or three amino acids bound to *p*‐nitrophenylalanine. Hydrolysis of these substrates produces *p*‐nitroaniline, which can be spectrophotometrically followed at 410 nm. Surprisingly, he hydrolysed product of **1** was not observed, but we did observe (slow) hydrolysis of substrate **2** (Figure S7). Although the LmrR_V15/M89HQAla_Zn^II^ enzymes were active on substrate **2**, no *p*‐nitroaniline was produced when LmrR with Zn(NO_3_)_2_ was added. Therefore, the hydrolysis of **2** is thought to rely on the Zn^II^ containing active site in LmrR_HQAla.

**Figure 3:**
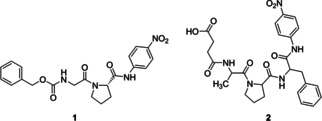
Substrates used for the amide‐bond hydrolysis reaction.

Finally, the catalytic activity of Cu^II^/HQAla‐containing artificial metalloenzymes was evaluated in the vinylogous Friedel‐Crafts alkylation reaction of 5‐methoxy‐1*H*‐indole (**4**) with 1‐(1‐methyl‐1*H*‐imidazol‐2‐yl)but‐2‐en‐1‐one (**3**; Scheme 1a),[^6e^, ^20^] and the 1,4‐addition of water to α,β‐unsaturated 2‐acyl pyridine **6** resulting in the corresponding β‐hydroxy ketone product **7** (Scheme 1b).[^6f^, ^14^, ^21^]

**Scheme 1:**
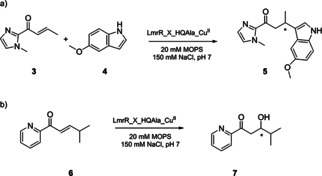
a) Artificial metalloenzyme‐catalysed vinylogous Friedel‐Crafts reaction and b) water addition. Reactions were run in 3‐(*N*‐morpholine)propanesulfonic acid (MOPS) buffer (20 mM, 150 mM NaCl, pH 7.5). After incubation of Cu(NO_3_)_2_ with the protein for 2 h, the relevant substrates were added, and reactions were run at 4 °C for 24 h.

Both reactions were carried out using 9 mol % of Cu(NO_3_)_2_ (90 μM) with a small excess of LmrR_V15HQAla or LmrR_M89HQAla (112.5 μM of the monomer). The uncatalysed Friedel–Crafts alkylation proceeds with 4 % conversion, whereas the unbound Cu(NO_3_)_2_ catalysed this reaction to give 99 % conversion (Table 1, entries 1, 2). The reaction catalysed by LmrR_V15HQAla_Cu^II^ yielded similar results to those with Cu(NO_3_)_2_ in solution, suggesting that although Cu^II^ binds to HQAla in the hydrophobic pore (Figure 2a), the metal catalyst is situated too far from the chiral scaffold to influence chirality of the reaction. (Table 1, entry 3). LmrR_M89HQAla_Cu^II^ gave rise to 25 % *ee* and 20 % conversion (Table 1, entry 4). While the conversion is lower than in the reaction catalysed by Cu(NO_3_)_2_, the enantioselectivity proves the benefit of the reaction occurring in a chiral scaffold.

**Table 1:** Results of the vinylogous Friedel‐Crafts reaction of **3** and **4** resulting in **5** and of the conjugate addition reaction of water to **6** resulting in **7**, both catalysed by LmrR_V15HQAla and LmrR_M89HQAla.

	Catalyst	Substrate	Product	Conv. [%]	*ee* [%]
1	–	**3**,**4**	**5**	4±2	–
2	Cu(NO_3_)_2_	**3**,**4**	**5**	99±1	–
3	LmrR_**V15HQAla**_Cu^II^	**3**,**4**	**5**	97±1	<5
4	LmrR_**M89HQAla**_Cu^II^	**3**,**4**	**5**	20±2	25±3
5	–	**6**	**7**	11±3	–
6	Cu(NO_3_)_2_	**6**	**7**	84±7	–
7	LmrR_**V15HQAla**_Cu^II^	**6**	**7**	77±8	<5
8	LmrR_**M89HQAla**_Cu^II^	**6**	**7**	20±5	51±10

[a] Typical conditions: 9 mol% Cu(H_2_O)_6_(NO_3_)_2_ (90 μM) loading with 1.25 equiv. LmrR variant in 20 mM MOPS buffer, 150 mM NaCl, pH 7.0 for 1 day at 4 °C. All data are the average of two independent experiments, each carried out in duplicate, reporting the average conversion/ee with their respective standard deviation.

The water addition (Scheme 1b) was catalysed by Cu(NO_3_)_2_ to give 84 % conversion (Table 1, entry 6). With Cu^II^ bound to LmrR_V15HQAla, a small decrease in activity was observed while the enantioselectivity stayed very low (Table 1, entry 7). Similar to the Friedel‐Crafts alkylation, LmrR_M89HQAla_Cu^II^ lowered the yield, but concomitantly increased the *ee* (51 %) (Table 1, entry 8).

Overall, our novel artificial copper enzymes were successful in the catalysis of both studied reactions. Notably, similar trends were observed between the V15 and M89 mutants. With the LmrR_V15HQAla mutant, high conversions were obtained. These results can be explained either by ideal localisation of the central tryptophanes contributing to substrate binding, or by the HQAla moiety being more solvent exposed at this position. The latter seems more likely because of the low enantioselectivity of the reactions with this mutant. However, this hypothesis should ideally be confirmed with X‐ray structures. The low conversions with LmrR_M89HQAla suggest that HQAla incorporated at this position is less accessible, or does not form interactions favourable for catalysis, however this more constrained position does facilitate enantioselective product formation.

In summary, we have presented novel artificial metalloenzymes containing the noncanonical amino acid HQAla as metal‐binding moiety. The catalytic potential of these newly created artificial metalloenzymes was evaluated in various reactions. HQAla was successfully incorporated into the structure of LmrR at two different positions: V15 and M89. Both mutants of LmrR showed a good affinity for different metal salts, such as Cu(NO_3_)_2_, Zn(NO_3_)_2_ and Cp*RhCl_2_. The novel Zn^II^‐containing artificial metalloenzymes showed activity in the hydrolysis of peptide bonds, while Cu^II^‐containing variants showed activity in Friedel‐Crafts alkylation reaction of 5‐methoxyindole with α,β‐unsaturated‐2‐acyl imidazole and water‐addition reaction to α,β‐unsaturated 2‐acyl pyridine, although with low‐to‐moderate enantioselectivities. Overall, due to the high affinity of LmrR_HQAla towards the different metal salts and complexes, this artificial metalloenzyme can provide a platform for a range of different, currently unexplored, metal‐catalysed reactions. We believe that HQAla, together with other known metal‐binding ncAAs, open up the way for a variety of (new‐to‐nature) biotransformations.

## Conflict of interest

The authors declare no conflict of interest.

## Supporting information

As a service to our authors and readers, this journal provides supporting information supplied by the authors. Such materials are peer reviewed and may be re‐organized for online delivery, but are not copy‐edited or typeset. Technical support issues arising from supporting information (other than missing files) should be addressed to the authors.

Supplementary
