# Supplementary material for: A Hydroxyquinoline‐Based Unnatural Amino Acid for the Design of Novel Artificial Metalloenzymes
Source: Chembiochem. 2020 Jul 17;21(21):3077–81. doi: 10.1002/cbic.202000306 (PMC7689906; doi:10.1002/cbic.202000306)
Supplement: Supplementary file 1 — Supplementary [file CBIC-21-3077-s001.pdf]

ChemBioChem

Supporting Information

A Hydroxyquinoline-Based Unnatural Amino Acid for the Design of Novel Artificial Metalloenzymes

Ivana Drienovská[+], Remkes A. Scheele[+], Cora Gutiérrez de Souza, and Gerard Roelfes*

Supplementary Information

Table of contents

1. General remarks S3
2. Chemical synthesis of HQAla NMR and LC-MS spectra S3
3. Expression and Purification S10
4. SDS-PAGE S11
5. ESI-mass spectra S12
6. Analytical size-exclusion chromatography S14
7. UV-visible titrations S15
8. Catalysis – Hydrolysis S17
9. Catalysis – Friedel-Crafts reaction S20
10. Catalysis – Water addition S22
11. Supplementary references S24

1. General remarks

Chemicals were purchased from Sigma Aldrich, Acros or TCI chemicals and used without further purification. Column chromatography was performed on silica gel (Silicycle, 230–400 mesh). Solvents were removed in a rotary evaporator under reduced pressure at 40 °C unless otherwise noted. ^{1}H-NMR and ^{13}C-NMR spectra were recorded on a Varian 400 (400 and 100 MHz) in $CDCl_3$ or D_2O. Mass spectra (ESI-MS) were recorded on an Orbitrap XL (Thermo Fisher Scientific; ESI pos. mode). Conversions and enantiomeric excess determinations were performed by HPLC analysis (Chiralpak-AD column, Chiralpak AD-H) using UV-detection (Shimadzu SCL-10Avp). *E. coli* strains NEB5α and BL21 C43(DE3) (Stratagene) were used for cloning and expression. DNA sequencing was carried out by GATC Biotech (Berlin, Germany). FPLC columns were purchased from GE Healthcare.

2. Chemical synthesis of HQAla NMR and LC-MS spectra

Synthesis of 2-Amino-3-(8-hydroxyquinolin-3-yl)propanoic acid (HQAla)[1]

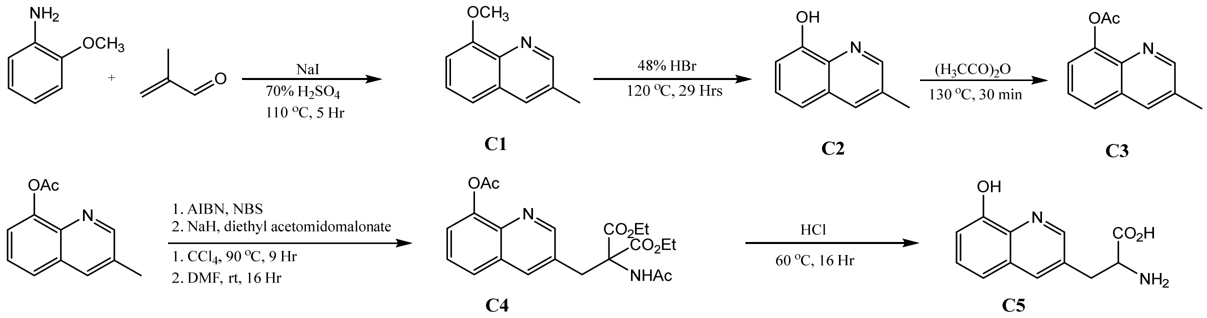

Scheme S1. Synthesis of HQAla.

The non-canonical amino acid used in this study, HQAla, was synthesized according to a previously reported method with a few minor improvements.[1] Full procedures as follows:

*8-Methoxy-3-methylquinoline (**C1**)*

Sodium iodide (0.15 g, 1.0 mmol), *o*-anisidine (8.2 mL, 73 mmol, distilled directly prior to addition) in sulfuric acid (26 mL 70%) were stirred in an oil bath at 110 °C. To this mixture, methacrolein (10 mL, 170 mmol) was added over 5 hours (2 mL/hour with an automatic syringe pump). After 1 h of additional stirring, the mixture was cooled to room temperature. Both the liquid phase and the solid black residue were found to contain our product. The liquid phase was poured onto 200 mL 1 M NaOH solution, filtered and extracted with 3 x 100 mL CH_2Cl_2. The solid black residue was dissolved in CH_2Cl_2 (stirring for 1 hour), after which both CH_2Cl_2 fractions were pooled, washed with water, dried over Na_2SO_4 and filtered. CH_2Cl_2 was evaporated under reduced pressure. The crude product was purified by flash column chromatography on silica (100 % CH_2Cl_2 → 95:5% CH_2Cl_2:MeOH) to afford 8-methoxy-3-methylquinoline (5.3 g, 36%) as a black solid. Optimized automatic flash column chromatography conditions; CH_2Cl_2:MeOH (20' 100:0, 10' 95:5, 5' 90:10, 5' 90:10). Analytical data were in accordance with those previously published.[1] ^{1}H NMR (400 MHz, $CDCl_3$) δ 8.82 (s, 1H), 7.95 (s, 1H), 7.45 (t, *J* = 8.0 Hz, 1H), 7.33 (d, *J* = 8.3 Hz, 1H), 7.02 (d, *J* = 1.1 Hz, 1H), 4.09 (s, 3H), 2.53 (s, 3H).

*3-Methylquinolin-8-ol (**C2**)*

To 8-methoxy-3-methylquinoline (5.3 g, 34 mmol), HBr was added (48%, 53 mL in acetic acid). The mixture was heated to 120 °C and heated under reflux for 29 hours. The mixture was neutralized with 3 M sodium hydroxide. The mixture was filtered and extracted with CH_2Cl_2. The organic layer was dried over Na_2SO_4 and concentrated *in vacuo*. The crude product was purified using flash column chromatography on silica (100% CH_2Cl_2). The product eluted first, after which the combined fractions were concentrated *in vacuo* to afford 3-methylquinoline (1.1 g, 23%) as a brown solid. Optimized automatic flash column chromatography conditions; CH_2Cl_2:MeOH (CH_2Cl_2:MeOH (30' 100:0, 20' 95:5, 10' 90:10, 5' 90:10). Analytical data were in accordance with those previously published.[1] ^{1}H NMR (400 MHz, $CDCl_3$) δ 8.64 (d, *J* = 2.1 Hz, 1H), 7.96 (s, 1H), 7.44 (t, *J* = 7.9 Hz, 1H), 7.28 (s, 1H), 7.14 (d, *J* = 7.6 Hz, 1H), 2.54 (s, 3H).

*3-Methylquinoline-8-yl acetate (**C3**)*

3-methylquinolin-8-ol (1.1 g, 6.9 mmol) was dissolved in 16.5 mL acetic anhydride and stirred for 30 min at 130 °C. The reaction mixture was cooled to room temperature and concentrated *in vacuo* at 60 °C. The residue was dissolved in 40 mL EtOAc and washed with 3 x 50 mL saturated $NaHCO_3$. The organic layer was dried over Na_2SO_4 and concentrated *in vacuo* to afford 3-methylquinoline-8-yl acetate (0.9 g, 79%) as a brown solid. Analytical data were in accordance with those previously published.[1] ^{1}H NMR (400 MHz, $CDCl_3$) δ 8.92 – 8.59 (m, 1H), 7.97 (s, 1H), 7.75 – 7.62 (m, 1H), 7.51 (t, *J* = 7.9 Hz, 1H), 7.39 (d, *J* = 7.5 Hz, 1H), 2.67 – 2.47 (m, 6H).

*2-Acetamido-2-((8-acetoxyquinoline-3-yl)methyl)malonate (**C4**)*

To a dry 100 mL flask 3-methylquinoline-8-yl acetate (1.6 g, 8 mmol), *N*-bromosuccinimide (1.6 g, 8.9 mmol), azobisisobutyronitril (142 mg, 8.9 mmol) and CCl_4 (67 mL) were added. The mixture was heated under reflux for 15 h at 90 °C. The reaction was followed over time by ^{1}H-NMR and additional NBS or AIBN was added if needed. The mixture was dissolved in 200 mL CH_2Cl_2 and washed with 3 x 100 mL saturated $NaHCO_{3\ (aq)}$. The organic layers were dried over Na_2SO_4 and concentrated *in vacuo* to give the crude brominated product. The crude product was immediately used for subsequent alkylation. To a dry 100 mL flask diethyl acetamidomalonate (1.7 g, 8.0 mmol), sodium hydride (0.3 g, 8.0 mmol, 60% in mineral oil) and dry DMF (40 mL) were added, stirring for 30 minutes at 0 °C under nitrogen atmosphere. The crude product was dissolved in dry DMF (8 mL) and very slowly added to the mixture of diethyl acetamidomalonate, sodium hydride in DMF at 0 °C, after which it was warmed to room temperature and stirred for 1 hour. The mixture was diluted with EtOAc (200 mL), washed with 10% aqueous sodium thiosulphate (3 x 100 mL) after which the organic layer was dried over Na_2SO_4 and concentrated *in vacuo*. The crude product was purified by flash column chromatography on silica (70:30% Pentane:EtOAc → 100% EtOAc. The product eluted after several column volumes of 100% EtOAc to afford 2-acetamido-2-((8-acetoxyquinoline-3-yl)methyl)malonate (0.4 g, 11%) as a white solid. Optimized automatic flash column chromatography conditions: heptane:EtOAc (20' 70:30, 15' 50:50, 10' 50:50). Analytical data were in accordance with those previously published. ^{1}H NMR (400 MHz, $CDCl_3$) δ 8.59 (d, *J* = 2.1 Hz, 1H), 7.84 (s, 1H), 7.66 – 7.61 (m, 1H), 7.53 (t, *J* = 7.8 Hz, 1H), 7.43 (d, *J* = 7.4 Hz, 1H), 6.58 (s, 1H), 4.30 (m, *J* = 7.1, 1.9 Hz, 4H), 3.87 (s, 2H), 2.50 (s, 3H), 2.06 (s, 3H), 1.31 (t, *J* = 7.1 Hz, 6H).

*2-Amino-3-(hydroxyquinolin-3-yl)propanoic acid dihydrochloride (**C5**)*

2-acetamido-2-((8-acetoxyquinoline-3-yl)methyl)malonate (0.4 g, 0.7 mmol) was dissolved in 12 M HCl (6 mL) and heated to reflux overnight. The mixture was concentrated *in vacuo* to afford 2-amino-3-(hydroxyquinolin-3-yl)propanoic acid dihydrochloride as a yellow powder (220 mg, 99%). Analytical data were in accordance with those previously published.[1] LC-MS (ESI) Mass$_{calc}$ for $C_{12}H_{12}N_2O_3$ ($M+H^+$) 233.08, Mass$_{obs}$ 233.07 ^1H NMR (400 MHz, D_2O) δ 8.91 – 8.84 (m, 2H), 7.69 – 7.57 (m, 2H), 7.38 – 7.31 (m, 1H), 4.30 – 4.20 (m, 1H), 3.51 (d, *J* = 6.9 Hz, 2H).

C1 Methoxy-3-methylquinoline

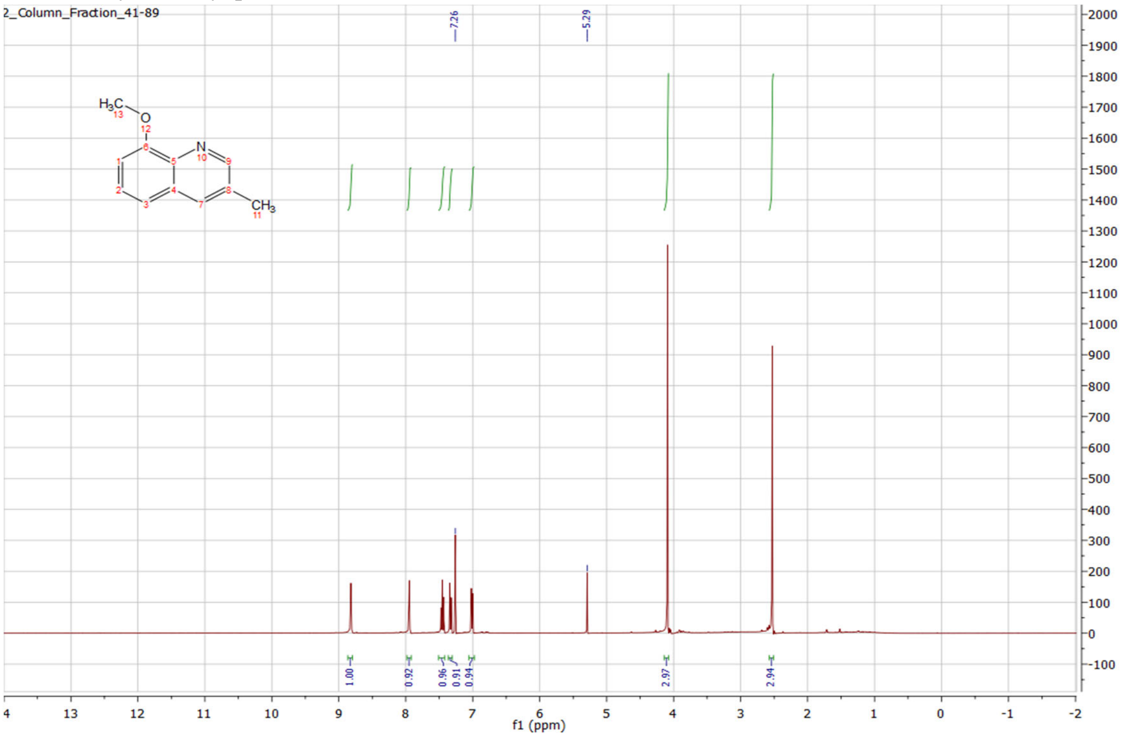

C2 2-Acetamido-2-((8-acetoxyquinoline-3-yl)methyl)malonate

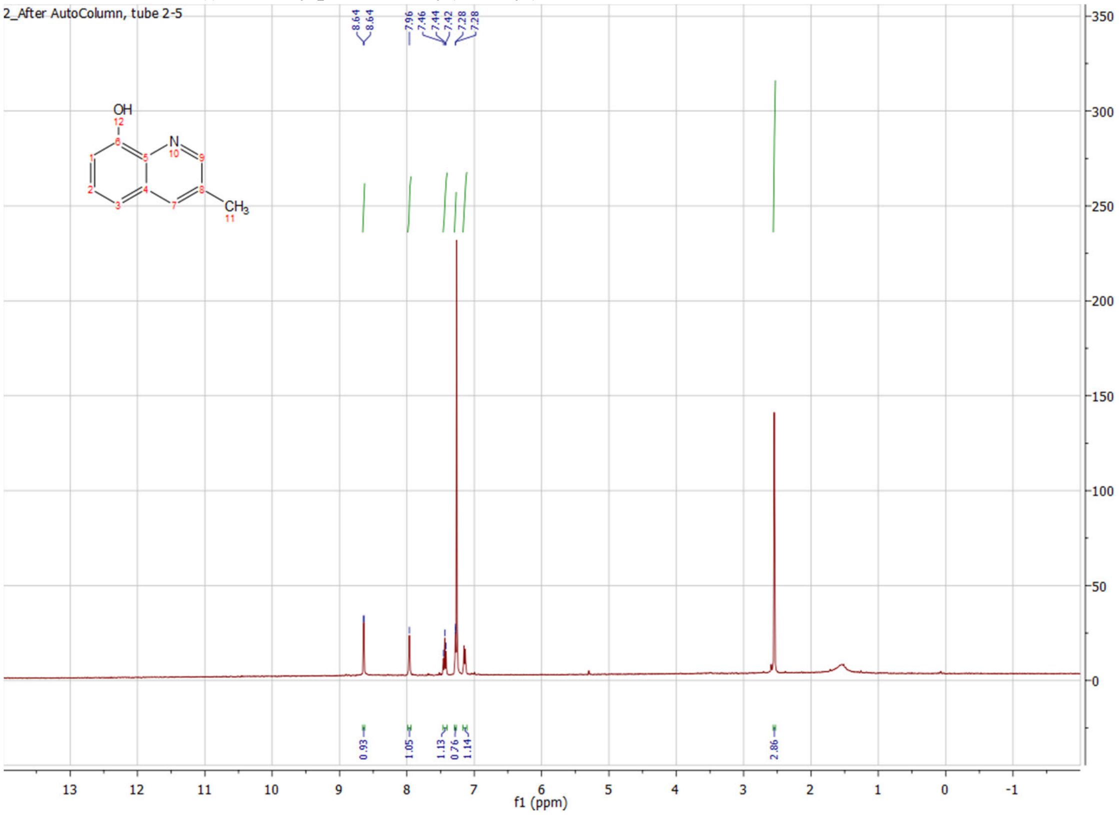

C3 3-Methylquinoline-8-yl acetate

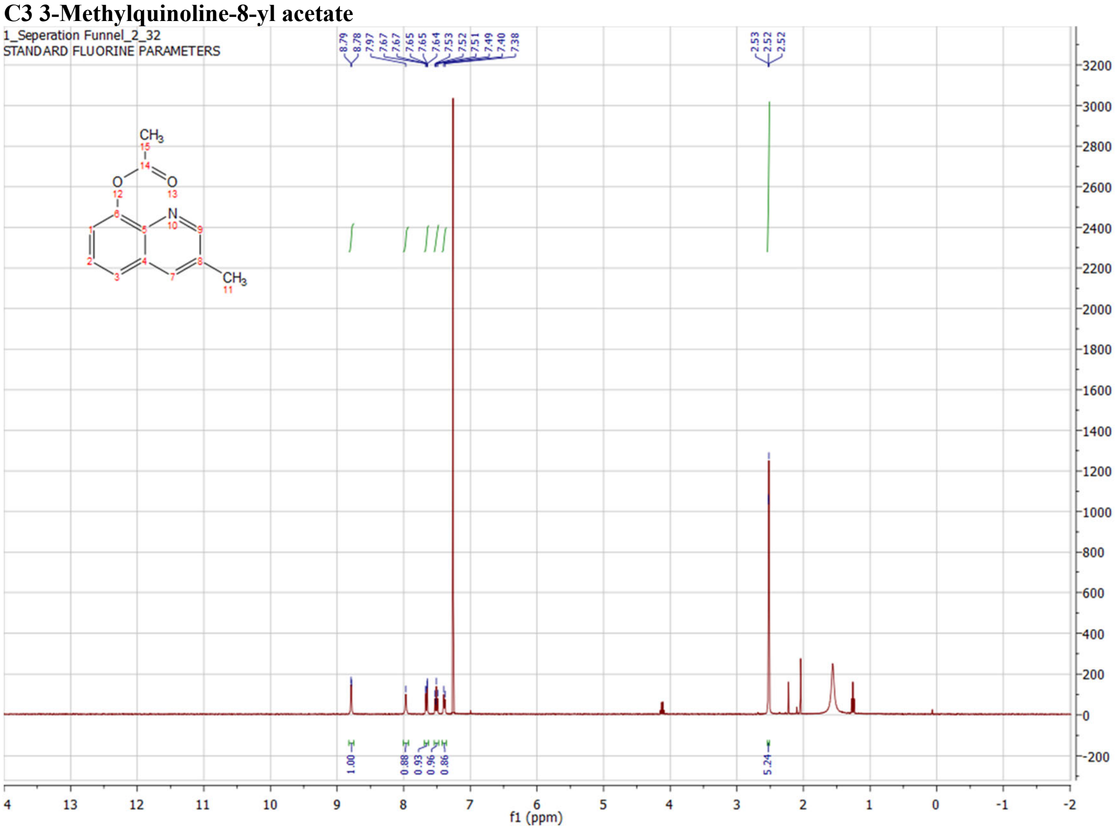

C4 2-Acetamido-2-((8-acetoxyquinoline-3-yl)methyl)malonate

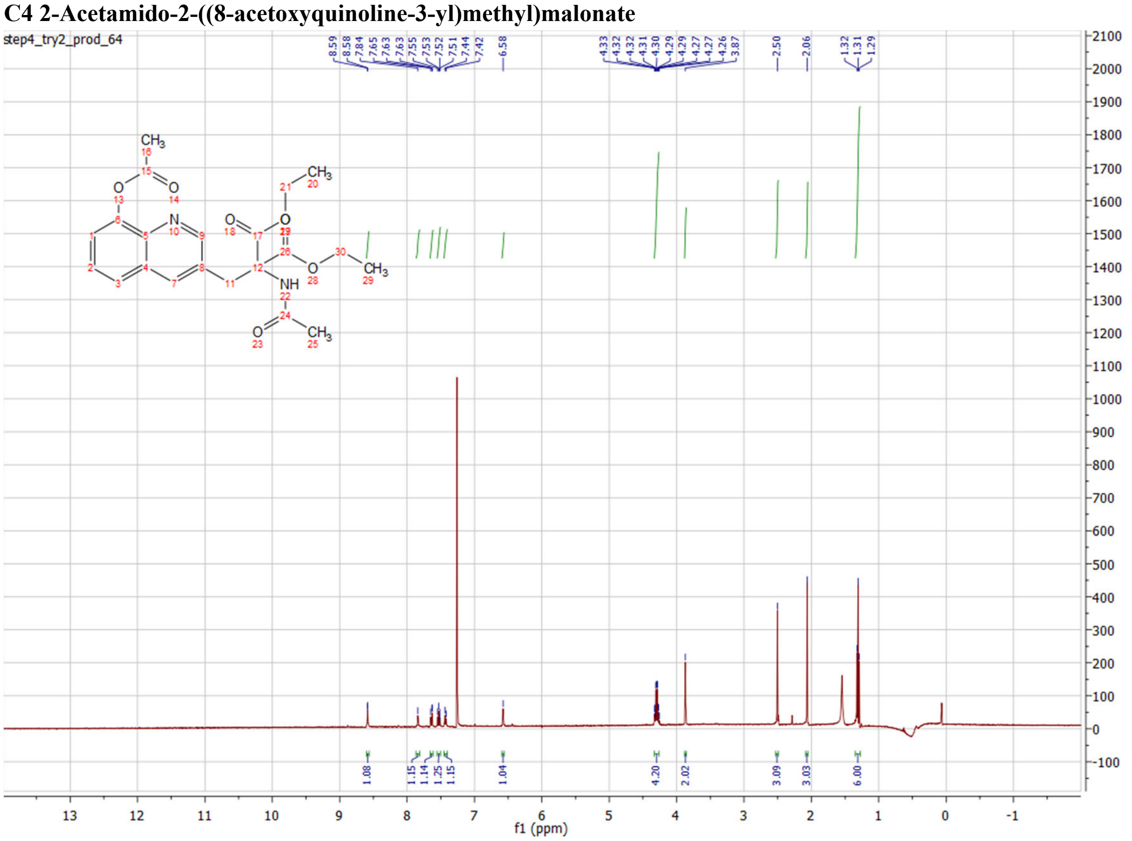

C5 2-Amino-3-(hydroxyquinolin-3-yl)propanoic acid dihydrochloride

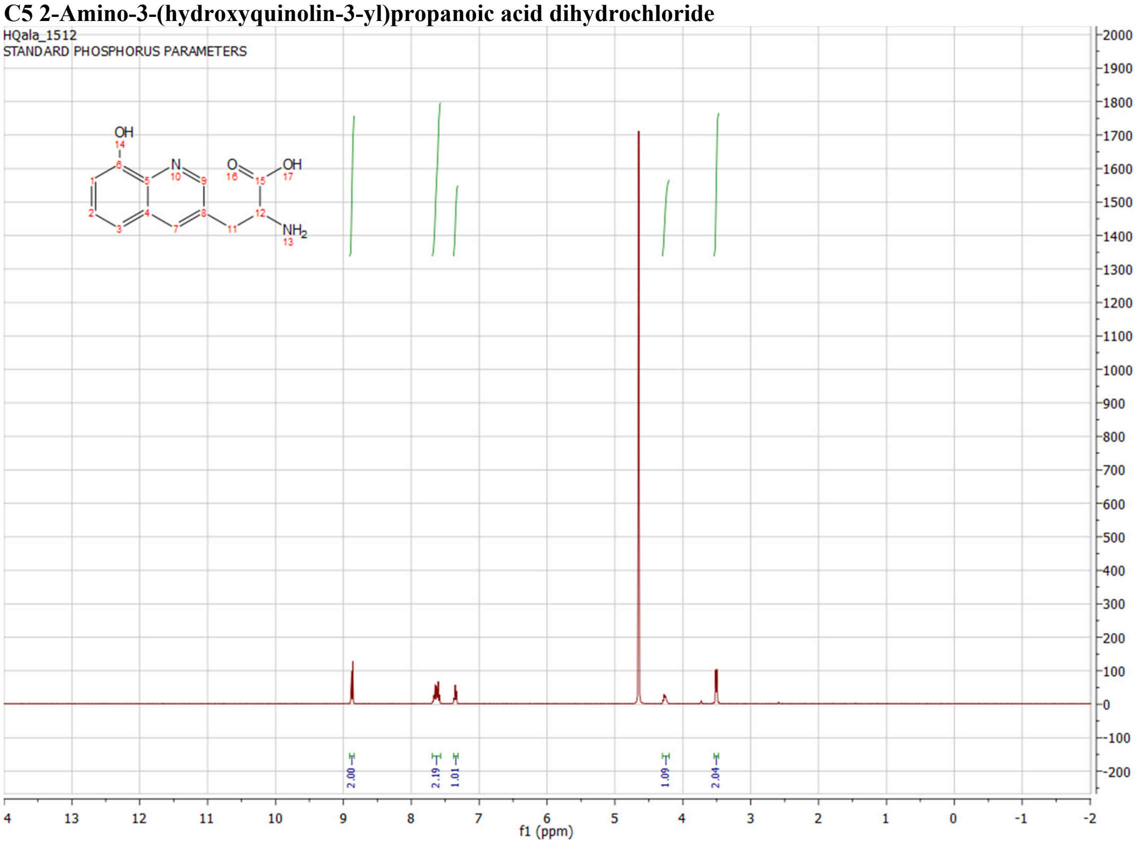

Analytical LC-MS (ESI) for **C5** ($C_{12}H_{12}N_2O_3$) ($M+H^+$) Mass$_{Calc}$ 233.08. Mass$_{Obs.}$:233.07

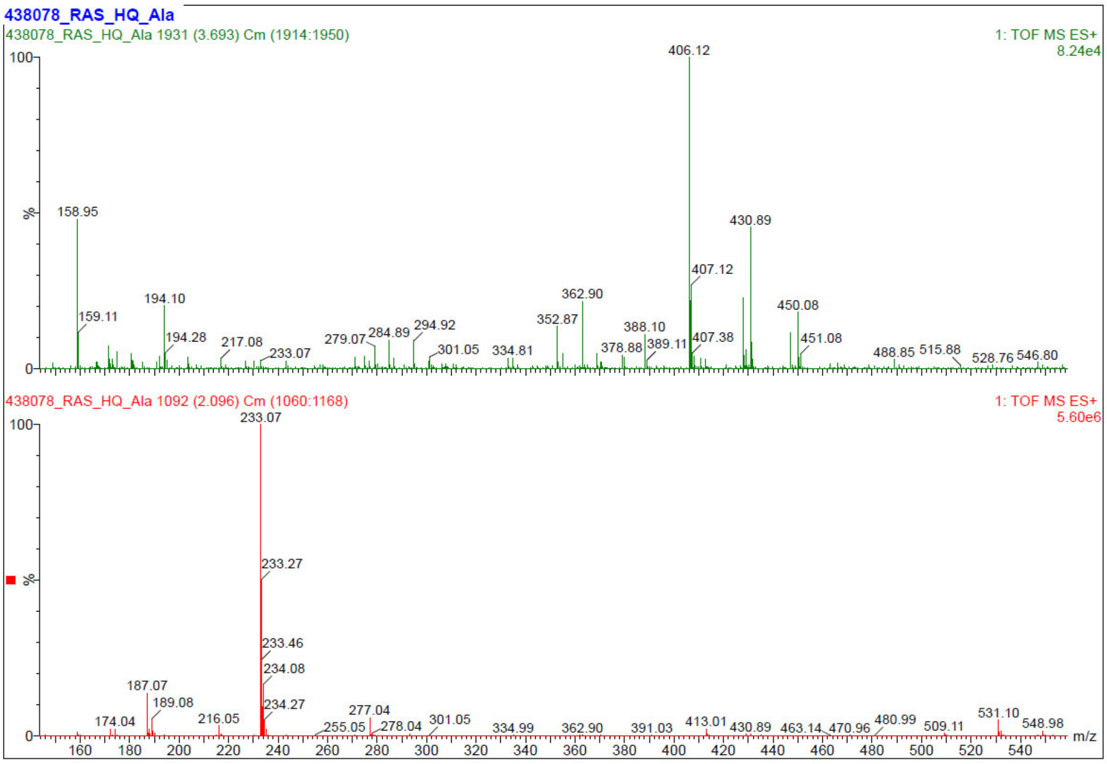

2. Expression and purification

The plasmids pEVOL-HQAla (a gift from P. G. Schultz at The Scripps Research Institute, USA) and pET17b_LmrR_V15TAG/M89TAG were cotransformed into *E. coli* BL21 C43(DE3) and a single colony was used to inoculate an overnight culture of 10 mL of fresh LB medium containing 100 µg/mL of ampicillin and 34 µg/mL of chloramphenicol. 1 mL (500x dilutions) of overnight culture was used to inoculate 500 mL of fresh LB medium containing 100 µg/mL of ampicillin 34 µg/mL of chloramphenicol. When the culture reached an optical density at 600 nm of 0.8–0.9, expression was induced with isopropyl β-D-1-thiogalactopyranoside (IPTG) (final concentration 1 mM) and L-Arabinose (final concentration 0.02%) and HQAla (60 mg, final concentration 0.2 mM). Expression was done overnight at 30 °C. Cells were harvested by centrifugation (6000 rpm, JA10, 20 min, 4 °C, Beckman), resuspended in washing buffer (50 mM NaH_2PO_4, 150 mM NaCl, pH 8.0) and sonicated (75% (200W) for 8 min (10 sec on, 15 sec off). The lysed cells were incubated with DNAseI (final concentration 0.1 mg/mL with 10 mM $MgCl_2$) and PMSF solution (final concentration 0.1 mM) for 1 hour at 30 °C. After centrifugation (15000 rpm, JA-17, 1h, 4 °C, Beckman), the supernatant was loaded on a Strep-Tactin column and incubated for 1 h. The column was washed with 3 x 1 column volume (CV) of resuspension buffer (same as wash buffer used before), and eluted with 6 x 0.5 CV of resuspension buffer containing 2.5 mM desthiobiotin. The fractions were analyzed on a 12% polyacrylamide SDS-Tris Tricine gel followed by Coomassie staining (InstantBlue, Expedeon). The concentration of the proteins was determined by using the calculated extinction coefficient ε_{280} = 25440 M^{-1} cm^{-1} (per monomer) (Protparam, Expasy server). In order to use proteins in the catalysis, they were dialysed against MOPS buffer (20 mM MOPS, 150 mM NaCl, pH 7.0) overnight at 4 °C.

4. SDS-PAGE

Figure S1. 12% Tricine SDS-PAGE analysis after Strep-Tag purification. Figure legend: L - Marker (SigmaMarker™ low range, 6500-66000 Da), FT – Flow through, W - wash fraction, E - elution fraction. Gels were stained with InstantBlueTM (Expedeon).

LmrR_V15HQAla

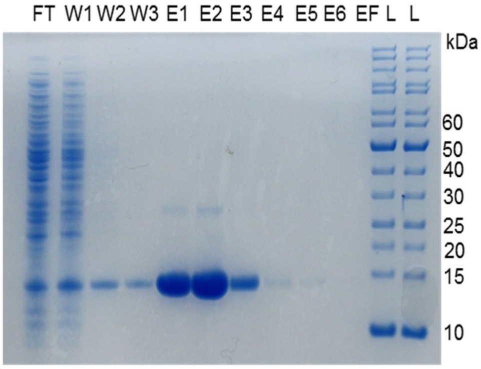

LmrR_M89HQAla

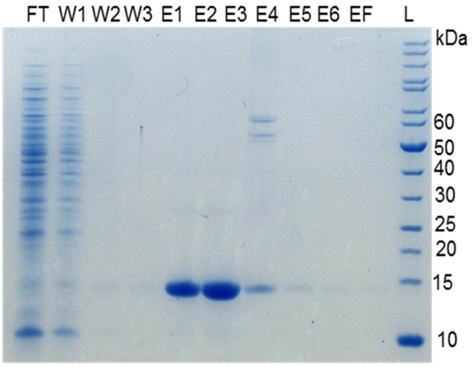

5. ESI-mass spectra

High-resolution mass spectrometry (LTQ Orbitrap XL, Thermo Fisher) was used to determine the exact mass of the prepared proteins, using electrospray ionization as the ion source. The protein mass was calculated using the expasy peptide mass calculator (**http://web.expasy.org/peptide_mass/**).

LmrR (-Met) = 14970.80 MW

(M)**G**AEIPKEMLRAQTNVILLNVLKQGDNYVYGIIKQVKEASNGEMELNEATLY TIF**D**RLE**Q**DGIISSYWGDESQGGRRKYYRLTEIGHENMRLAFESWSRVDKIIENL EANKKSEAIK**SRGGSGGWSHPQFEK**

ΔHQala-Valine = (233.07 - 117.15) = 115.92 MW

ΔHQala-Methionine = (233.07 - 149.21) = 83.86 MW

Figure S2. Electrospray ionization (ESI) mass spectra of LmrR variants**.**

LmrR_V15HQAla $Mass_{Calc}$ (-Met) 15086.72. $Mass_{Obs.}$:15086.60.

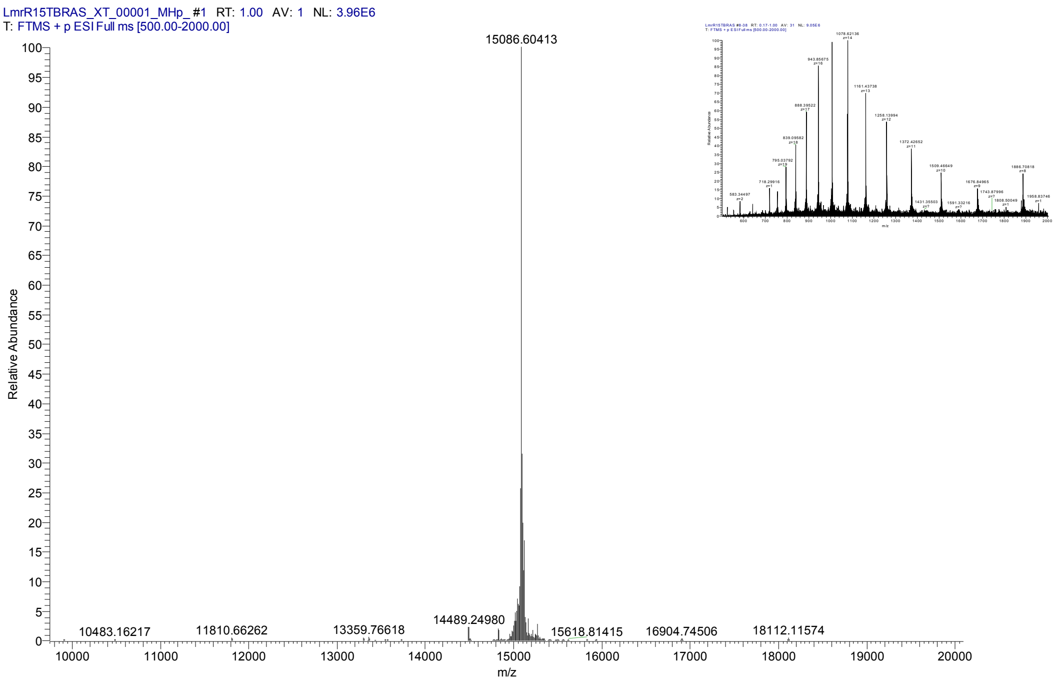

LmrR_M89HQAla Mass$_{Calc}$ (-Met):15054.66, Mass$_{Obs.}$:15053.61

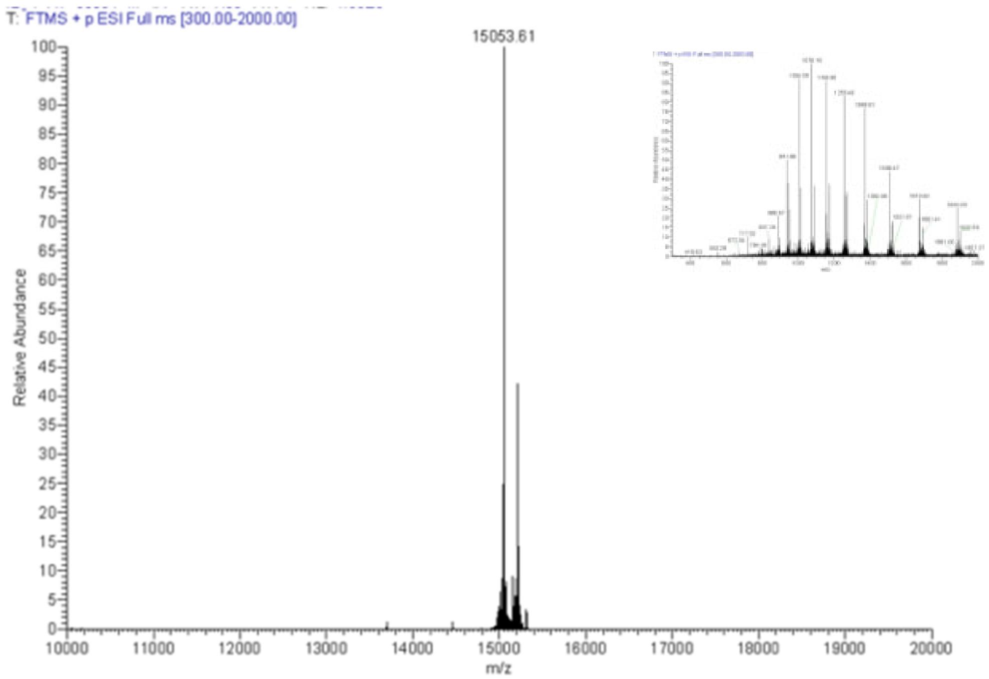

6. Analytical size-exclusion chromatography

Analytical size-exclusion chromatography was performed on a Superdex 75 10/300 GL (GE Healthcare). 100 µL of the sample was injected using 20 mM MOPS, 150 mM NaCl pH 7.0, as buffer (flow 0.5 mL/min). The column was calibrated using the standard Gel Filtration LMW Calibration Kit of GE Healthcare.

Figure S3. Analytical size exclusion chromatography (Superdex-75 10/300 GL)

LmrR_V15HQAla

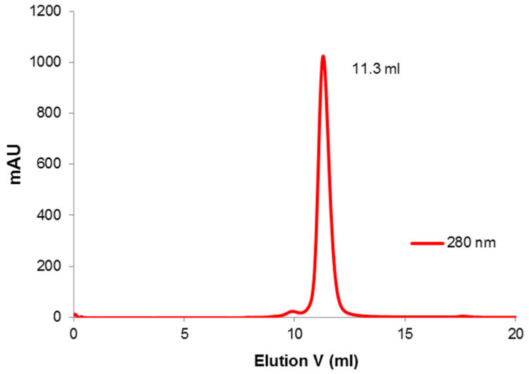

LmrR_M89HQAla

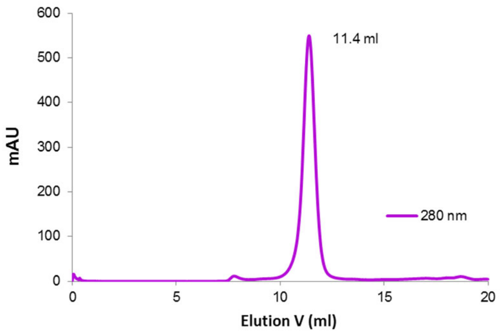

7. UV-visible titrations

LmrR with and without HQAla were diluted to 20 µM (10 µM for the LmrR_M89_HQAla titration with $Cu(NO_3)_2$, all calculated on the monomer) in MOPS buffer (20 mM MOPS, 150 NaCl, pH 7.0) of which 300 µL was transferred to 1 mL quartz cuvettes. The absorption spectrum was measured from 700-220 nm in a JASCO UV-VIS V-660 spectrophotometer with data intervals of 1 nm. Transition metal salts were added to the cuvette in steps of 0.25 equivalent to the protein monomer until 2 equivalents of the metal salt were added.

Figure S4. UV-visible titrations with a) $Zn(NO_3)_2$ and b) full spectrums (220-700 nm) of titrations with both $Zn(NO_3)_2$ and $Cu(NO_3)_2$ with additional zoomed inserts from 250-450 nm.

a)

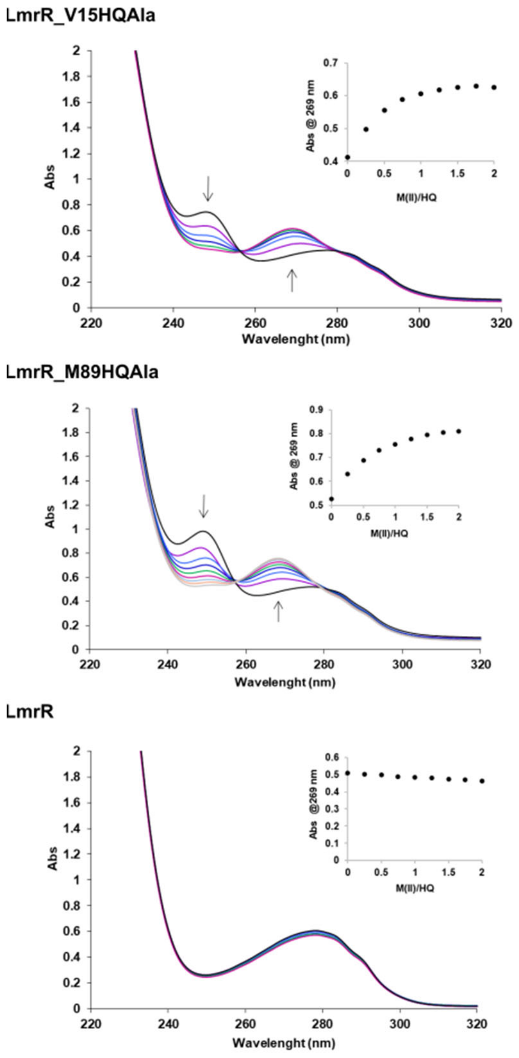

b)

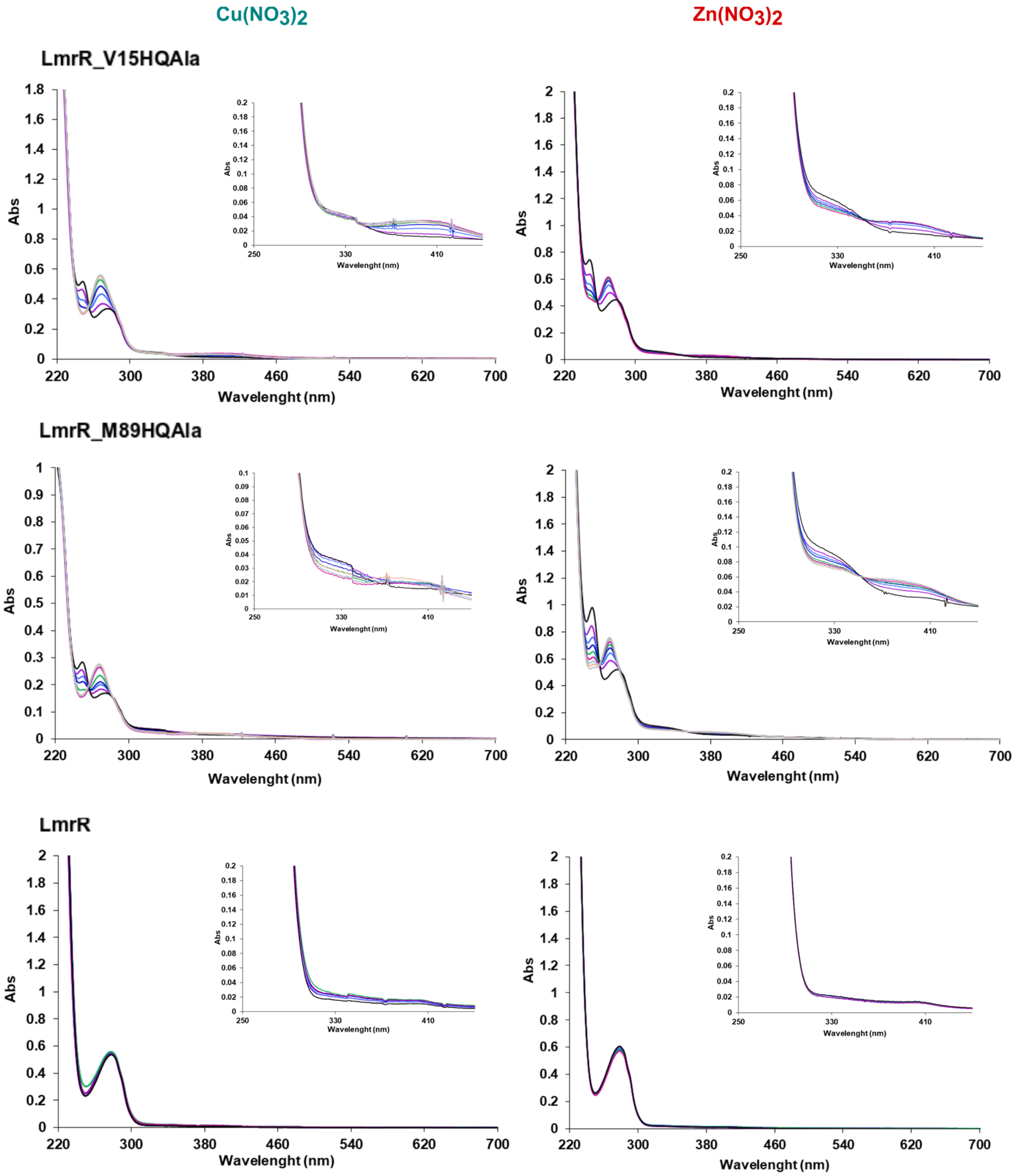
Cu(NO3)2
Zn(NO3)2
LmrR_V15HQAla
LmrR_M89HQAla
LmrR
Abs
Wavelenght (nm)

Figure S5. UV-visible titrations with rhodium complexes.

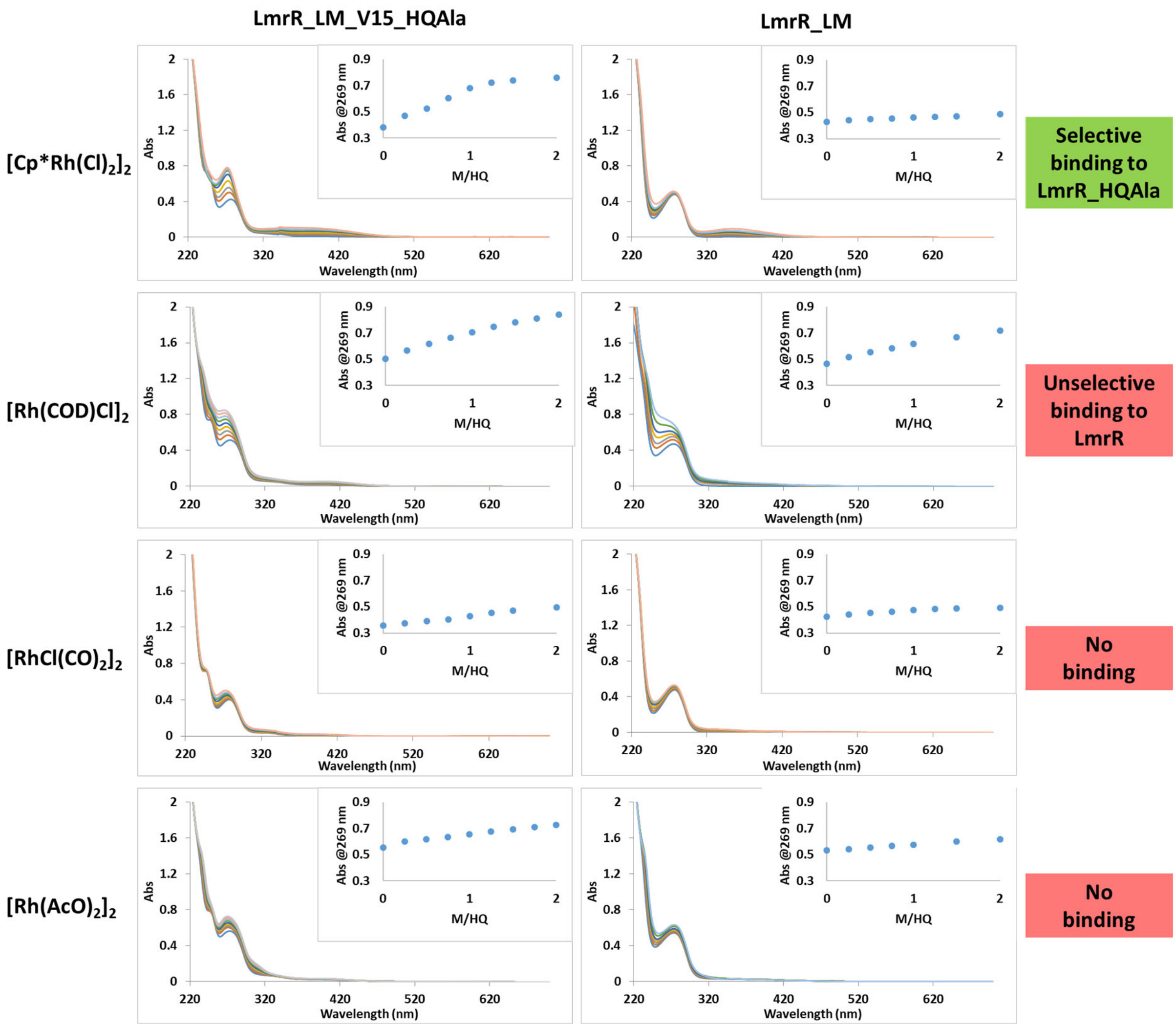

8. Catalysis – Hydrolysis

Representative procedure for hydrolysis

The hydrolysis reaction was monitored spectroscopically for 60 min at 400 nm (*p*-nitrophenylacetate, *p*-nitrophenylbutyrate, *p*-nitrophenylphosphate, **3-5**), or 410 nm (substrates **1, 2**) using a Jasco V-660 spectrophotometer. Reactions were carried out in 300 μL solutions (buffer 20 mM MOPS, 150 mM NaCl, pH 7.0) in 0.5 mL quartz cuvettes (path length 1 cm). The final concentration of the protein in the solution was 20 μM (reaction with substrates **3-5**) or 5 μM (reaction with substrates **1**, **2**) (concentration of the monomer). The reactions were started by the addition 10 μL solution of substrate (final concentration 3-5: 1 mM, 1,2: 0.25 mM). The absorbance data were converted to concentration of the product using the extinction coefficient of the product (*p*-nitrophenolate=12800 $M^{-1}cm^{-1}$ at 400 nm, *p*-nitrophenylalanine=8,800 $M^{-1}cm^{-1}$ at 410 nm). Experiments were performed as two independent measurements.

Figure S6. Substrates used for the hydrolysis reactions.

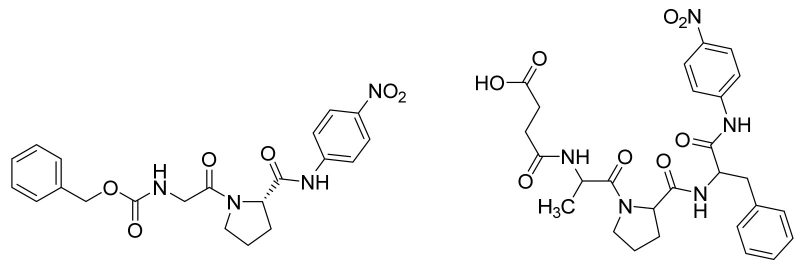

1 **2**

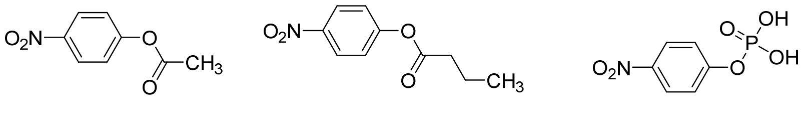

3 **4** **5**

Figure S7. a) Hydrolysis of the substrate **3**, uncatalyzed and catalyzed with LmrR_V15/M89_HQAla_Zn^{II}. The initial reaction rates are presented. **b)** Hydrolysis of the substrate **3**, uncatalyzed and catalyzed with LmrR and its mutant variants, with or without Zn^{II} bound. The concentration of the substrate **3** is 1 mM with 20 µM concentration of the enzymes (in monomer). The conditions were the same also in (a). **c)** Hydrolysis of the substrate **2,** uncatalyzed and catalyzed with LmrR and its mutant variants, with or without Zn^{II} bound. The concentration of the substrate **2** is 250 µM with 5 µM concentration of the enzymes (in monomer).

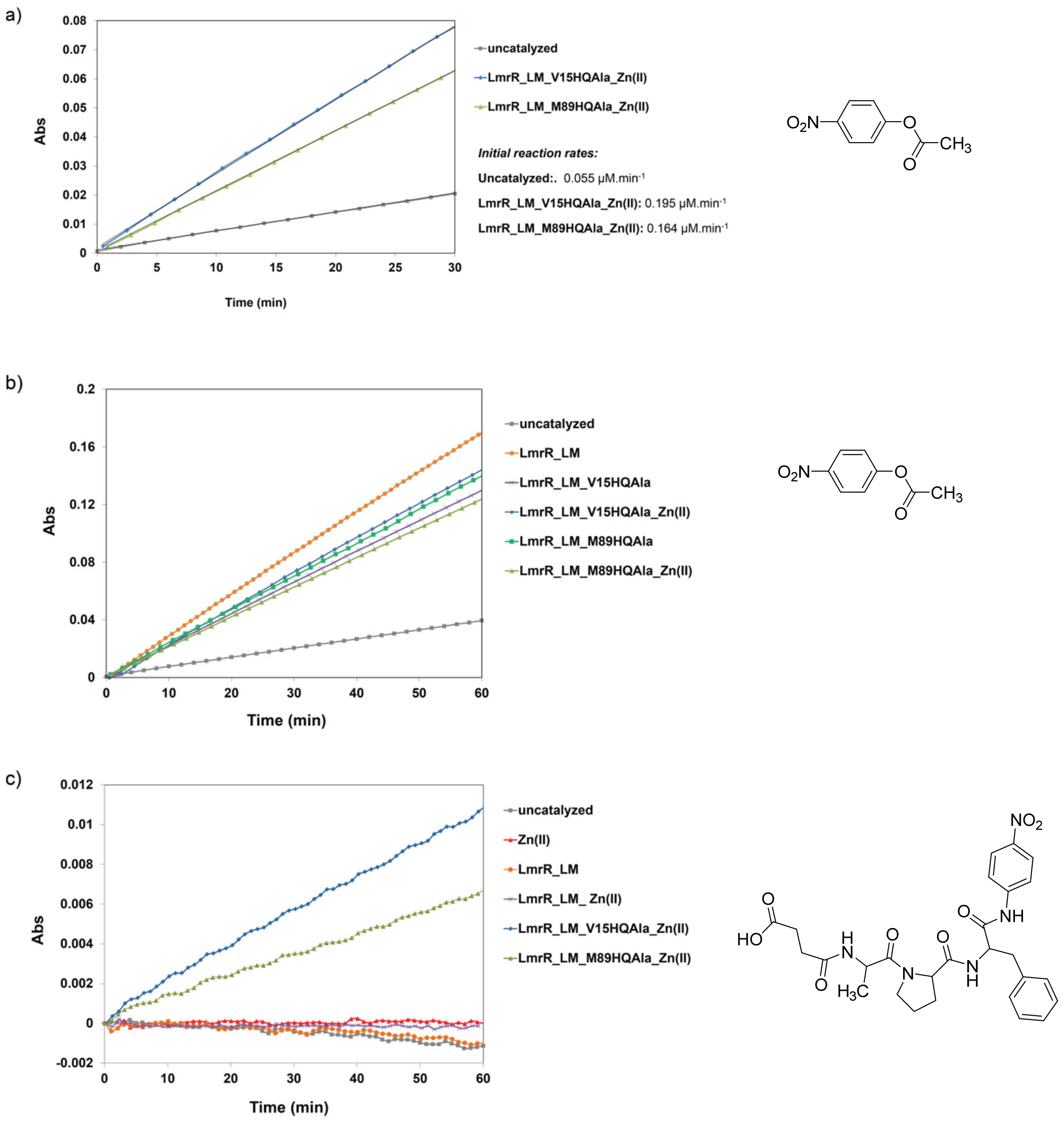

9. Catalysis – Friedel-Crafts reaction

Representative procedure for Friedel-Crafts reaction

The catalytic solution was prepared by combining $Cu(H_2O)_6(NO_3)_2$ (90 µM, 9 % catalyst loading) in MOPS buffer (20 mM MOPS, 150 mM NaCl, pH 7.0) with 1.25 equivalents of LmrR_V15/M89_HQAla (112.5 µM in monomer) to a final volume of 280 µL. After incubation, 10 µL of a fresh stock solution of 5-methoxy-1*H*-indole in CH_3CN (final concentration 2.5 mM) and 10 µL of solution of 1-(1-methyl-1H-imidazol-2-yl)but-2-en-1-one in MOPS/CH_3CN was added (final concentration 1 mM). The reaction was mixed by continuous inversion for 1 day at 4 °C. The product was extracted with 3 x 1 mL of diethyl ether, the organic layers were dried on Na_2SO_4 and evaporated under reduced pressure. The product was redissolved in 150 µl of a heptane:propan-2-ol mixture (10:1) and the conversion and enantiomeric excess were determined using HPLC (Chiralpak-AD n-heptane:iPrOH 90:10, 1 mL/min).

Figure S8. Chiral HPLC traces of results of the Friedel-Crafts reaction.

Catalyst: LmrR_V15HQala_Cu^{II}

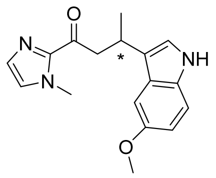

<Chromatogram>

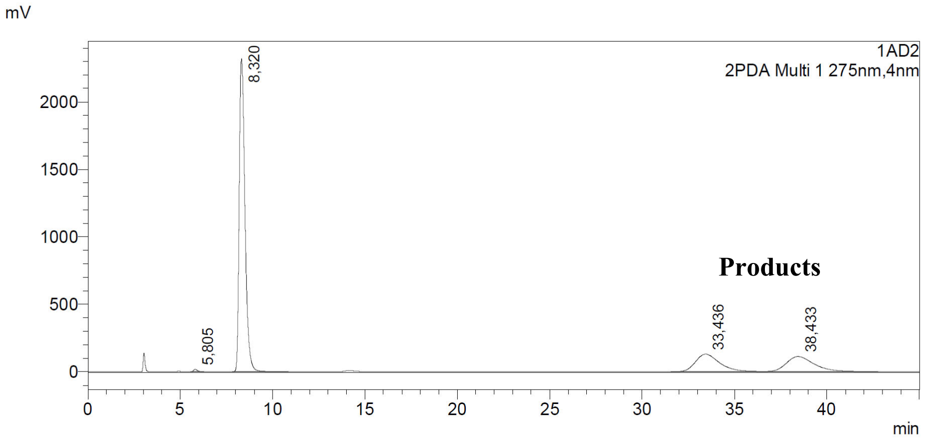

<Peak Table>

AD2

Peak#	Ret. Time	Area	Height	Conc.	Unit	Mark	Name
Total							

PDA Ch1 275nm

Peak#	Ret. Time	Area	Height	Conc.	Unit	Mark	Name
1	5,805	249535	18470	0,000			
2	8,320	50286026	2318682	0,000		S	
3	33,436	11915251	129806	0,000			
4	38,433	11821603	112070	0,000		SV	
Total		74272415	2579028				

Catalyst: LmrR_M89HQala_Cu^{II}

<Chromatogram>

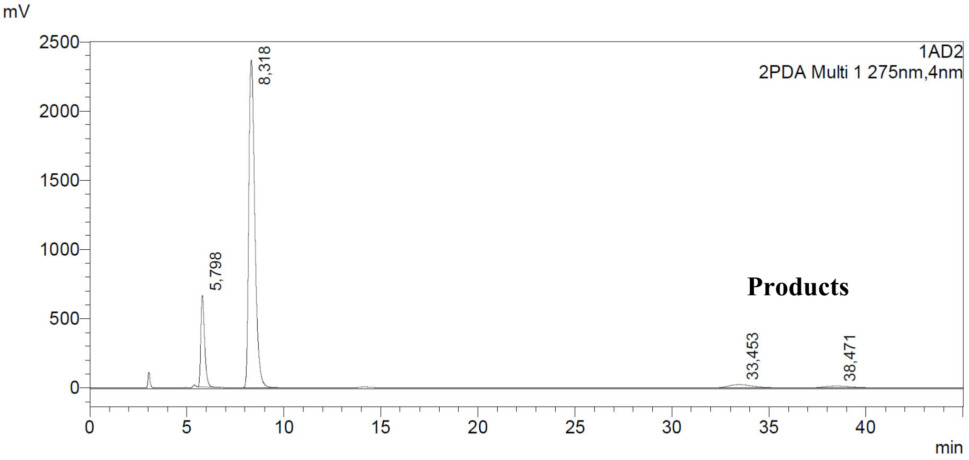

<Peak Table>

AD2

Peak#	Ret. Time	Area	Height	Conc.	Unit	Mark	Name
Total							

PDA Ch1 275nm

Peak#	Ret. Time	Area	Height	Conc.	Unit	Mark	Name
1	5,798	9051494	661322	0,000			
2	8,318	51502981	2366402	0,000		S	
3	33,453	2147118	24009	0,000		V	
4	38,471	1384113	13639	0,000		S	
Total		64085707	3065372				

Catalyst: $Cu(NO_3)_2$

<Chromatogram>

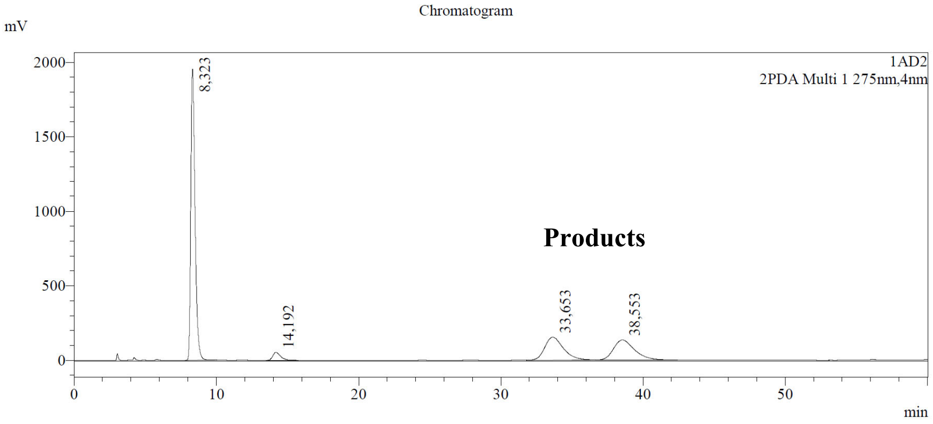

<Peak Table>

Peak Table

AD2

Peak#	Ret. Time	Area	Height	Conc.	Unit	Mark	Name
Total							

PDA Ch1 275nm

Peak#	Ret. Time	Area	Height	Conc.	Unit	Mark	Name
1	8,323	40630562	1954709	0,000		S	
2	14,192	1982817	52721	0,000			
3	33,653	14280861	154791	0,000		V	
4	38,553	14093455	135050	0,000		V	
Total		70987696	2297273				

10. Catalysis – Water-addition reaction

Representative procedure for water-addition reaction

Procedure similar to the one of Friedel-Crafts alkylation. Changes included use of a 290 µL solution of LmrR_V15/M89_HQAla (final conc. 112.5 µM of monomer) with $Cu(NO_3)_2$ (10 µl in MiliQ grade water, final conc. 90 µM). After incubation, the substrate (*E*)-4-methyl-1-(pyridin-2-yl)pent-2-en-1-one (10 µL in CH_3CN/MOPS buffer, final conc. 1 mM) was added. Conversion and enantiomeric excess were determined using HPLC (Chiralpak-ADH n-heptane:iPrOH 99:1, 0.5 mL/min for 120 minutes). Substrates and products of tested reactions have been synthetized according to previously published procedures.[2]

Figure S9. Chiral HPLC traces of results of the water addition reaction.

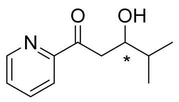

Catalyst: LmrR_V15HQala_Cu^{II}

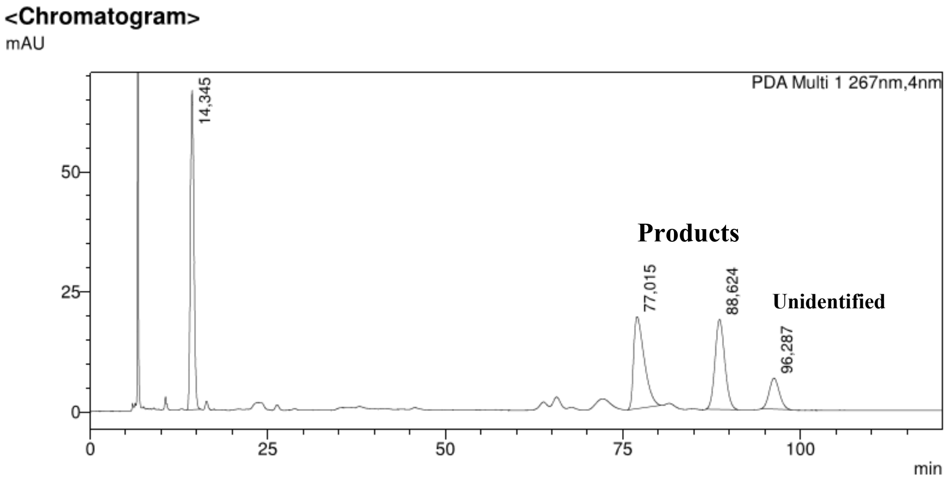

<Peak Table>

PDA Ch1 267nm

Peak#	Ret. Time	Area	Height	Conc.	Unit	Mark	Name
1	14,345	2339024	66510	0,000			
2	77,015	1989505	19152	0,000			
3	88,624	1795183	18756	0,000			
4	96,287	651347	6493	0,000			
Total		6775058	110911				

Catalyst: LmrR_M89HQala_Cu^{II}

<Chromatogram>
mAU

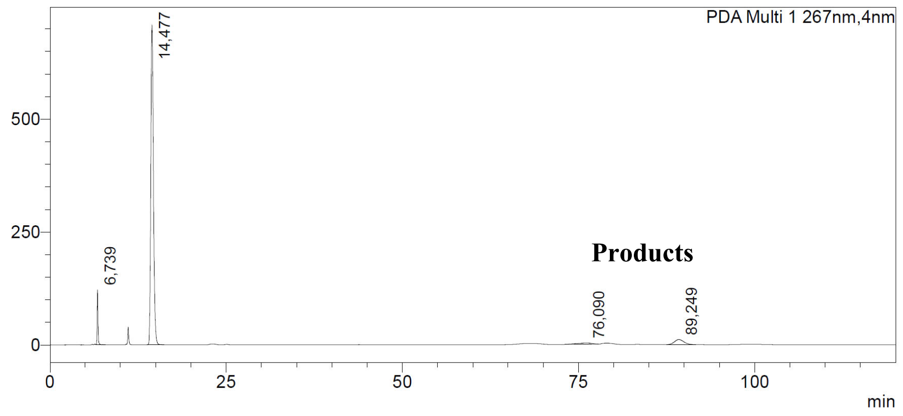

<Peak Table>

PDA Ch1 267nm

Peak#	Ret. Time	Area	Height	Conc.	Unit	Mark	Name
1	6,739	1024850	122105	0,000		SV	
2	14,477	18431618	707691	0,000			
3	76,090	421393	3154	0,000			
4	89,249	1012586	11108	0,000			
Total		20890446	844058				

Catalyst: $Cu(NO_3)_2$

<Chromatogram>
mAU

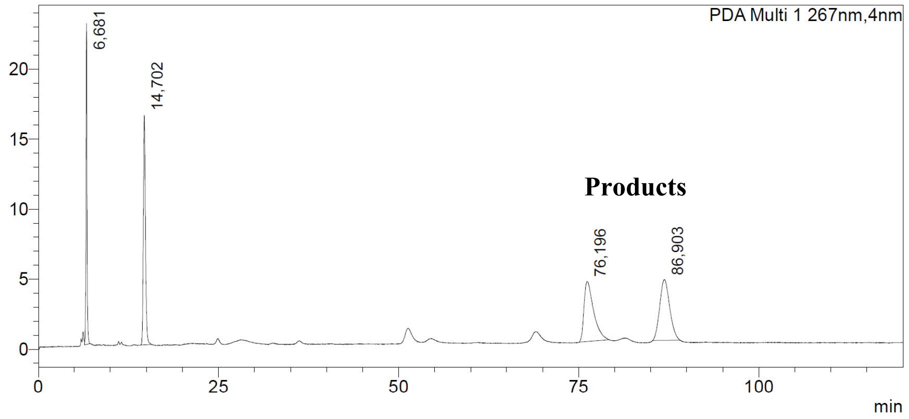

<Peak Table>

PDA Ch1 267nm

Peak#	Ret. Time	Area	Height	Conc.	Unit	Mark	Name
1	6,681	217126	22924	15,914		V	
2	14,702	333355	16410	24,433			
3	76,196	395904	4286	29,018			
4	86,903	417952	4337	30,634			
Total		1364337	47957				

11. Supplementary references

[1] H. S. Lee, G. Spraggon, P. G. Schultz, F. Wang, *J. Am. Chem. Soc.* **2009**, *131*, 2481–2483.

[2] J. Bos, G. Roelfes, *Curr. Opin. Chem. Biol.* **2014**, *19C*, 135–143.
